# Chloroplast Genome Sequences of Seven Strains of the Bloom-Forming Raphidophyte *Heterosigma akashiwo*

**DOI:** 10.1128/genomeA.01030-17

**Published:** 2017-10-12

**Authors:** Sergio Seoane, Kiwamu Hyodo, Shoko Ueki

**Affiliations:** a Department of Plant Biology and Ecology, University of the Basque Country (UPV/EHU), Lejona, Spain; b Institute of Plant Science and Resources, Okayama University, Okayama, Japan

## Abstract

We report here the complete chloroplast genome sequences of seven strains of the bloom-forming raphidophyte *Heterosigma akashiwo*. These ~160-kb sequences contain 124 protein-, 6 rRNA-, and 34 tRNA-coding sequences. Notable sequence variations were observed among these seven sequenced and two previously characterized strains.

## GENOME ANNOUNCEMENT

*Heterosigma akashiwo* is a eukaryotic, unicellular, bloom-forming alga that belongs to the family *Raphidophyceae*. It has been identified in the Pacific and Atlantic oceans and in both the northern and southern hemispheres, thus spanning a wide climate range from arctic to tropic (1–12). Because its blooms may exert a negative impact on local ecosystems, its population dynamics in the environment are of great interest and importance. Establishing strain markers will allow the monitoring of geographical and temporal dynamics of the species. We previously sequenced whole-mitochondrial genomes of four strains of this species and identified a hypervariable protein-coding sequence associated with the geographic origins of the strains (13–15).

Here, as a part of our continuous effort to establish strain-specific markers for *H. akashiwo*, we sequenced full-length chloroplast DNA (ptDNA) of seven *H. akashiwo* strains with different geographic origins: CCAP934/8 (Puget Sound, WA, USA), EHUSP1 (Bay of Biscay, Spain), CCAP934/4 (Tampa Bay, FL, USA), CCMP2274 (San Francisco Bay, CA, USA), CCMP3374 (East Greenwich, RI, USA), HaFk01 (Fukuoka, Japan), and CCMP1596 (Narragansett, RI, USA). Total DNA was extracted from *H. akashiwo* cells using the DNeasy blood and tissue kit (Qiagen). The libraries were sequenced with the Illumina MiSeq platform, and the obtained paired-end reads were mapped to the previously published sequences, *H. akashiwo* ptDNA of NIES293 (EU168190) and CCMP452 (EU168191) (16), using Burrows-Wheeler alignment software. The mapped reads were extracted using Samtools software and assembled using the Platanus software (17). The obtained contigs were aligned with the reference sequences, and the corresponding segments in the references were replaced with the contigs. The process was repeated up to three times to obtain the final assemblages, and the accuracy of the assembly was validated by mapping the reads on the obtained sequences (18). Protein-coding sequence (open reading frame) prediction and gene annotation were performed with ORFfinder and database searches with BLASTx; tRNAs were predicted by tRNA-scan server, and rRNA coding sequences were identified by a database search with BLASTn.

The lengths of the ptDNAs of strains CCAP934/8, EHUSP01, CCAP934/4, CCMP2274, CCMP3374, HaFk01, and CCMP1596 were 159,918 bp, 160,150 bp, 160,099 bp, 159,321 bp, 160,152 bp, 159,492 bp, and 159,691 bp, respectively, and G+C contents were 30.5% for all the strains. Twenty-four genes related to photosynthesis (two *cfxQ*s,* psaA*,* psaB*,* psaC*, *psaD*,* psaF*,* psaL*,* psb28*, two *psbA*s, *psbB*, two *psbC*s, two* psbD*s,* psbE*,* psbH*, *psbV*, two *rbcL*s, *ycf3*,* ycf34*, and *ycf35*) were identified in each strain, as reported previously for strains CCMP452 and NIES293. Similarly, 34 previously known tRNA genes, including 2 pseudogenes, were identified in all strains. As previously reported, all strains possessed ~22-kb invert repeats, and the strains CCAP934/8, CCMP2274, and HaFk01 contained an ~8.0-kb inversions, flanked between the *tyrC* and *psb28* genes, compared to those of strains EHUSP01, CCAP934/4, CCMP3374, and CCMP1596. The parts of the *H. akashiwo* ptDNA sequences, between 17.5 and 18 kb and 66 and 66.5 kb, showed notable polymorphisms, which may be utilized as strain-specific markers.

### Accession number(s).

The sequences reported here were deposited in DDBJ/EMBL/GenBank under the accession numbers LC269918 (CCAP934/8), LC269919 (EHUSP01), LC269920 (CCAP934/4), LC269921 (CCMP2274), LC269922 (CCMP3374), LC269923 (HaFk01), and LC269924 (CCMP1596).
